# High-efficiency and low efficiency roll-off near-infrared fluorescent OLEDs through triplet fusion†
†Electronic supplementary information (ESI) available: Compound characterisation data, crystallographic data, UPS spectra, EL spectra of device E, emission spectra of NSeD in toluene, phosphorescence spectra of Ga_2_(saph)_2_q_2_. CCDC 1402824. For ESI and crystallographic data in CIF or other electronic format see DOI: 10.1039/c5sc04685h


**DOI:** 10.1039/c5sc04685h

**Published:** 2016-01-19

**Authors:** Jie Xue, Chen Li, Lijun Xin, Lian Duan, Juan Qiao

**Affiliations:** a Key Lab of Organic Optoelectronics and Molecular Engineering of Ministry of Education , Department of Chemistry , Tsinghua University , Beijing 100084 , P R China . Email: duanl@mail.tsinghua.edu.cn ; Email: qjuan@mail.tsinghua.edu.cn

## Abstract

Though urgently needed, high-performance near-infrared organic light-emitting diodes (NIR-OLEDs) are still rare. NIR-OLEDs based on conventional NIR fluorescent materials usually suffer from low external quantum efficiencies (EQEs) because of the intrinsic obstacles according to the spin-statistics limit and energy-gap law. Herein, we realized high-efficiency and low efficiency roll-off fluorescent NIR-OLEDs through efficient triplet fusion of a bipolar host doped with a special naphthoselenadiazole emitter (4,9-bis(4-(2,2-diphenylvinyl)phenyl)-naphtho[2,3-*c*][1,2,5]selenadiazole, NSeD). Unlike typical NIR organic donor–acceptor (D–A) chromophores, NSeD features a non-D–A structure and a very large HOMO/LUMO overlap and displays a strong deep-red to NIR fluorescence and unique ambipolar character. The corresponding photoluminescence quantum efficiency of NSeD reaches 52% in solution and retains 17% in the blend film. The optimized NIR-OLEDs demonstrated a strong emission at 700 nm, a high maximum EQE of 2.1% (*vs.* the predicted theoretical maximum efficiency of 1.3%) and the EQE remained at around 2% over a wide range of current densities from 18 to 200 mA cm^–2^, which is amongst the highest performance for NIR-OLEDs based on organic fluorescent materials.

## Abstract 2

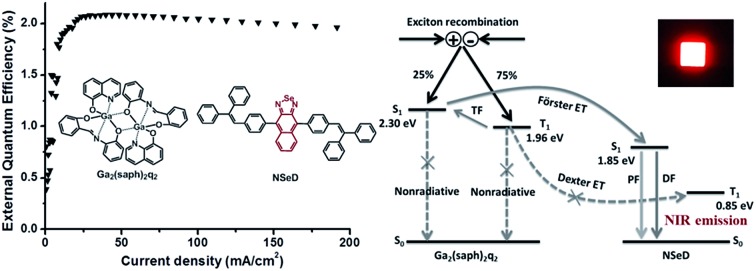
High-efficiency and low efficiency roll-off near-infrared fluorescent OLEDs are realized through effective triplet fusion of a bipolar host doped with a special non-donor–accepter naphthoselenadiazole emitter.

## Introduction

After decades of effort, organic light-emitting diodes (OLEDs) have been used to achieve great breakthroughs, with use in flat-panel displays, solid-state lighting and other applications especially in the visible region.^1^–^4^ Recently, the extension of visible light, near-infrared OLEDs (NIR-OLEDs) have aroused growing interest due to their potential application in night-vision and information-secured displays, optical telecommunication, phototherapy, *etc.*^5^–^9^ To date, several types of organic or metal-complex materials have been used as emitters in NIR-OLEDs, including fluorescent organic dyes with donor–acceptor (D–A) structures,^10^,^11^ transition-metal complexes,^12^ lanthanide complexes,^6^,^13^ and conjugated polymers.^14^–^16^ Amongst these materials, transition-metal complexes (*e.g.*, containing Pt^2+^, Os^3+^ or Ir^3+^) can exploit the normally non-radiative triplet excitons and therefore help realize high external quantum efficiencies (EQEs) for NIR-OLEDs.^12^,^17^–^23^ However, such high EQE values usually suffer from noticeable efficiency roll-offs with increasing current density, which could be mainly ascribed to the long lifetime of triplet excitons.^12^,^17^–^25^ In addition, the price and rarity of these noble metals would limit their mass production and future application.

Conventional organic fluorescent materials which have cost advantages show easily tunable emission through finely combining D–A chromophores;^11^ however, these D–A compounds usually exhibit low quantum efficiency in the NIR spectral range mainly because of the intrinsic limitation according to the energy-gap law, which describes an exponential increase in the non-radiative rate with a decrease of the energy gap.^26^,^27^ In addition, the limited overlap between the highest occupied molecular orbital (HOMO) and the lowest unoccupied molecular orbital (LUMO) in such D–A compounds results in a much lower radiative-transition rate.^26^,^28^ When further coupled with the upper limit of radiative singlet exciton ratio of 25% in conventional fluorescent OLEDs (FOLEDs), the corresponding NIR-OLEDs give very low EQEs (below 1%). Thus it is still highly challenging to realize highly efficient NIR-OLEDs based on conventional fluorescent materials, which would require innovative approaches to enhance not only the radiative-transition rate of NIR-emitting organic compounds but also the radiative exciton ratio of the corresponding OLEDs.

Approaches for harvesting the 75% triplet excitons of organic fluorescent materials are highly desired to enhance the radiative exciton ratio and thus enable high-efficiency and low-cost NIR-OLEDs. In 2014, Ma *et al.* proposed a hybridized local and charge-transfer (HLCT) state to employ high-lying triplet excitons and reported efficient NIR-OLEDs with a maximum EQE of 1.54% at around 700 nm and a high radiative exciton ratio of 48%.^29^ Recently, Wang *et al.* employed a thermally activated delayed fluorescence (TADF) emitter for efficient nondoped NIR-OLEDs with a maximum EQE of 2.1% and an emission at 710 nm.^30^ An alternative strategy to harness the triplet excitons of organic fluorescent materials involves triplet fusion (TF).^31^,^32^ The theoretical maximum singlet exciton production yield through TF is 50%, which would contribute a maximum radiative exciton ratio of up to 62.5%.^33^ In 2009, Kondakov *et al.* reported high-efficiency red FOLEDs through the TF of the host material achieving a high EQE of 11.3%.^33^ In 2013, Monkman *et al.* reported highly efficient green FOLEDs *via* ultrahigh-efficiency TF of a special anthracene/tribenzenamine emitter achieving a 6% EQE, which far exceeds the theoretical maximum EQE of 3.55%.^34^ Recently, Lu *et al.* reported a charge-transfer-featured naphthalimide derivative, which could harvest triplet excitons through TF and act as an efficient host for orange-red FOLEDs.^35^ To the best of our knowledge, there are no reports on efficient NIR-OLEDs through TF. Herein, we considered that TF could likewise provide an alternative strategy to realize highly efficient NIR-FOLEDs.

To enable highly efficient NIR-OLEDs through TF, the more feasible approach is *via* efficient TF of the host rather than direct TF from the emitter, since the triplet excitons of the NIR emitter may decay dominantly *via* non-radiative transition in accord with the energy gap law. In this work, we realized high-performance NIR-OLEDs *via* the high-efficiency TF of a bipolar host doped with a special naphthoselenadiazole emitter. The bipolar host material is bis(salicylidene-*o*-aminophenolato)-bis(8-quinolinoato)-bis-gallium(iii) [Ga_2_(saph)_2_q_2_], which was found to harvest triplet efficiently through TF and then transfer its singlet excitons to the dopant 4,9-bis(4-(2,2-diphenylvinyl)phenyl)-naphtho[2,3-*c*][1,2,5]selenadiazole (NSeD). Unlike typical D-A compounds, NSeD has a very large HOMO/LUMO overlap and displays strong deep-red to NIR fluorescence and the corresponding quantum efficiency reaches 52% in solution and retains 17% in the Ga_2_(saph)_2_q_2_:NSeD blend film. The optimized NIR-OLEDs without outcoupling enhancements achieved a strong NIR emission at 700 nm and a high EQE of up to 2.1% at 42 mA cm^–2^, which is far beyond the predicted theoretical maximum value of 1.3%. In particular, the EQEs remained at around 2% over a wide range of current densities from 18 to 200 mA cm^–2^.

## Results and discussions

**Fig. 1:**
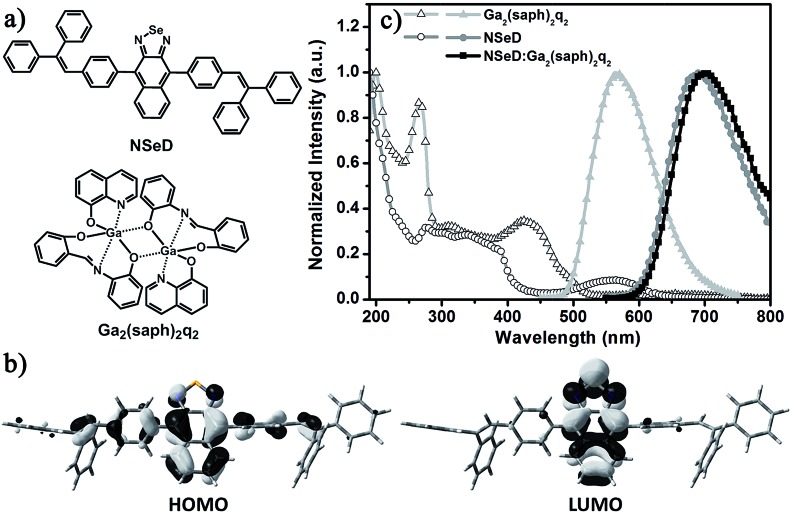
(a) Molecular structures of NSeD and Ga_2_(saph)_2_q_2_. (b) Isocontour plots of the frontier orbitals of NSeD. The isocontour value |*Ψ*| is 0.03. (c) The absorption (open symbol) and emission spectra (solid symbol) of Ga_2_(saph)_2_q_2_, NSeD and the 50 wt% Ga_2_(saph)_2_q_2_:NSeD films at 298 K.

Fig. 1 shows the molecular structures and the photophysical properties of the compounds used. The host material Ga_2_(saph)_2_q_2_ is a homemade binuclear gallium complex with an emission peak at 563 nm and bipolar character.^36^,^37^ Recently, it has proven to be an excellent host for a NIR-emitting iridium complex.^38^ In previous work, our group reported a series of naphtho[2,3-*c*][1,2,5]thiadiazole derivatives which possess both ambipolar transporting properties and high fluorescence quantum yields.^39^,^40^ Among these materials, 4,9-bis(4-(2,2-diphenylvinyl)phenyl)naphtho[2,3-*c*][1,2,5]thiadiazole (NTD) exhibits a highly efficient red emission. To tune the emission energy into the NIR region, NSeD was synthesized through replacing the sulfur atom of NTD with selenium.^41^–^43^ As depicted in the single crystal structure (Fig. S3†), NSeD is a nonplanar molecule based on naphtho[2,3-*c*][1,2,5]selenadiazole connected with bulky aryl substituents on both sides in the *trans* configuration. There are two sets of orientations for the naphthoselenadiazole core and each set of orientation possesses 50% occupancy. The crystal packing diagram (Fig. S4†) reveals that the weak C–H···N interactions (*D* = 2.847 Å, *θ* = 165.4°) are the main force maintaining molecular order; face-to-face π–π stacking interactions are negligible, which is beneficial to hinder the undesired photoluminescence self-quenching.

The electronic structures of NSeD were calculated using DFT calculations based on its single-crystal structure. Unlike typical organic D–A compounds, the HOMO of NSeD is mostly located on the naphthoselenadiazole core and has a small contribution from the aryl substituents directly connected with the naphthoselenadiazole core, while the LUMO is highly located on the naphthoselenadiazole core (Fig. 1b). Such large overlap between the HOMO and LUMO suggests that NSeD would possess desirable ambipolar character and enhanced fluorescence efficiency.^40^,^44^ In toluene solution, NSeD exhibits a strong deep-red emission at 670 nm (Fig. S5†) with a high photoluminescence quantum yield (PLQY) of 0.52. The net film of NSeD shows a marked bathochromic shift to 689 nm (Fig. 1c), while the PLQY falls to 0.13 because of aggregation-caused quenching (ACQ).^45^ When doped in Ga_2_(saph)_2_q_2_ at 50 wt%, the Ga_2_(saph)_2_q_2_:NSeD blend film gives a further bathochromic shift to 700 nm and a markedly improved PLQY of 0.17 due to the suppression of ACQ.

**Fig. 2:**
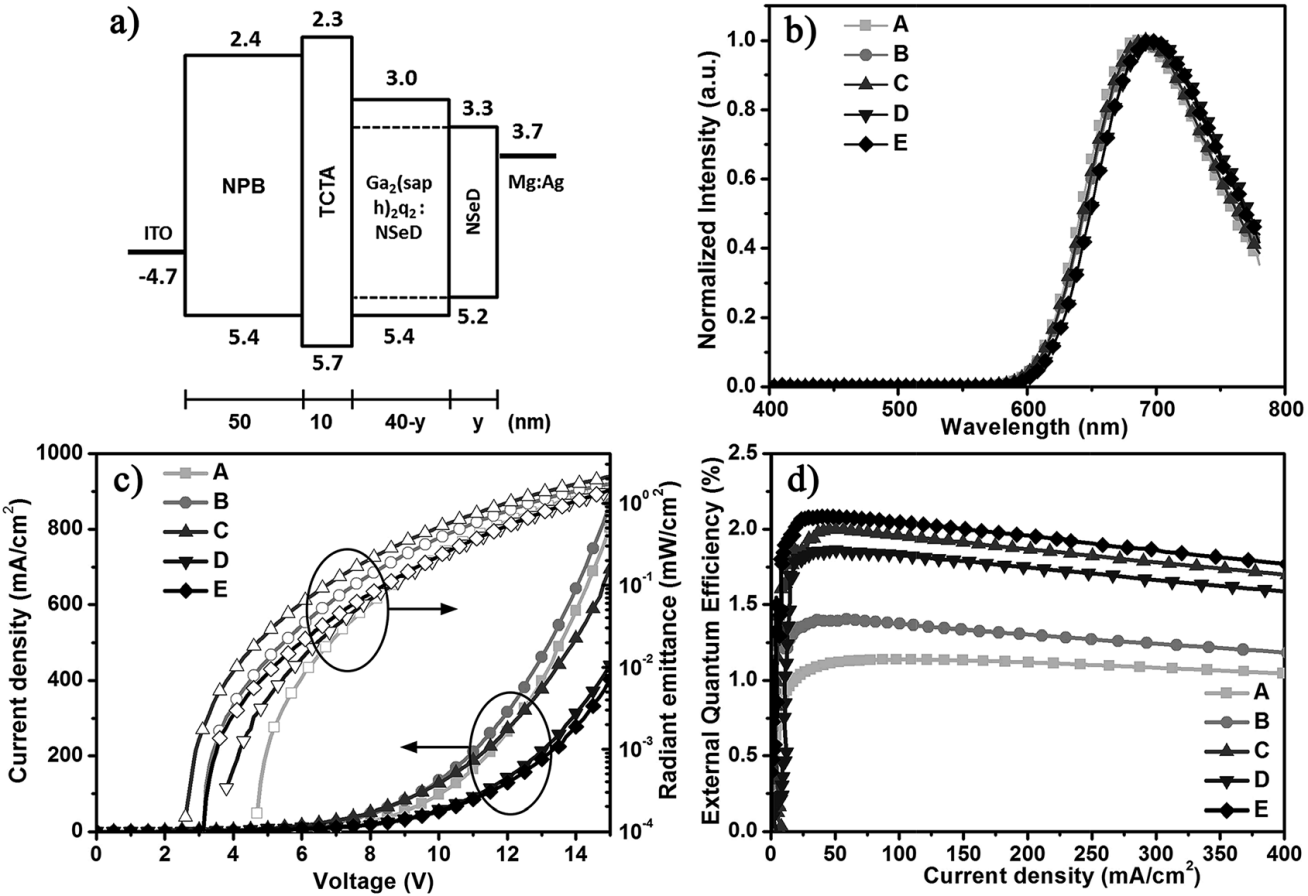
(a) The energy level diagram of the devices. (b) Electroluminescent spectra (at 8 V) of the devices with the architectures ITO/NPB (50 nm)/TCTA (10 nm)/Ga_2_(saph)_2_q_2_:NSeD (*x* wt%, 40-*y* nm)/NSeD (*y* nm)/Mg:Ag (150 nm) (device A: *x* = 100, *y* = 0, device B: *x* = 25, *y* = 0, device C: *x* = 50, *y* = 0, device D: *x* = 75, *y* = 0, device E: *x* = 50, *y* = 10). (c) Current density (*J*)–voltage (*V*)–radiant emittance (*R*) characteristics of the devices. (d) External quantum efficiency–current density characteristics of the devices.

Using Ga_2_(saph)_2_q_2_ as the host and NSeD as the dopant, we fabricated NIR-OLEDs with the device configurations of ITO/NPB (50 nm)/TCTA (10 nm)/Ga_2_(saph)_2_q_2_:NSeD (*x* wt%, 40-*y* nm)/NSeD (*y* nm)/Mg:Ag (150 nm) (device A: *x* = 100, *y* = 0, device B: *x* = 25, *y* = 0, device C: *x* = 50, *y* = 0, device D: *x* = 75, *y* = 0, device E: *x* = 50, *y* = 10), where ITO is indium tin oxide, NPB is *N*,*N*′-di(naphthalen-1-yl)-*N*,*N*′-diphenylbenzidine used as the hole transporting material, TCTA is tris(4-carbazoyl-9-ylphenyl)amine to lower hole transport and balance the charge carrier, and Mg:Ag is the cathode. The device architecture and the energy level diagram are depicted in Fig. 2a. The HOMO and LUMO levels of Ga_2_(saph)_2_q_2_ and NSeD were estimated from ultraviolet photoelectron spectroscopy (Fig. S6†) and by subtracting the optical energy gaps from the HOMO energies, respectively. The characteristics of these devices are listed in Table 1. Device A with pure NSeD showed an emission at 688 nm and a maximum EQE of 1.1% (Fig. 2d). Device B with 25 wt% NSeD achieved an improved EQE of up to 1.4%. As the NSeD concentration was increased in the emitting layer (EML), device C with 50 wt% NSeD exhibited a much better EQE of up to 2.0% with the emission red-shifted to 692 nm. However, the EQE of device D with 75 wt% NSeD decreased slightly to 1.9%, which is in accord with the relatively lower PLQY (0.14) of the 75 wt% Ga_2_(saph)_2_q_2_:NSeD blend film. Thus, device C with 50 wt% NSeD exhibited the best device performance. It is worth noting that device C showed the lowest operation voltage (Fig. 2c) with a low turn-on voltage of 2.6 V (at a radiant emittance of 1 mW m^–2^) and a maximum radiant emittance (*R*_max_) of 2127 μW cm^–2^ (at 15 V), which is among the highest values reported in NIR-FOLEDs peaking at around 700 nm.^10^,^46^–^48^

**Table 1:** Electroluminescence characteristics of the devices

Device	NSeD ratio [wt%]	*V*_on_ ^*a*^ [V]	*λ*_EL,max_ ^*b*^ [nm]	EQE^*c*^ [%]	*R* ^*d*^ [μW cm^–2^]
A	100	4.7	688	1.1, 1.1, 1.1	1477
B	25	3.2	692	1.4, 1.4, 1.3	1720
C	50	2.6	692	2.0, 2.0, 1.7	2127
D	75	3.8	700	1.9, 1.8, 1.7	1404
E	50	3.2	700	2.1, 2.1, 2.0	1451

^*a*^ *V*_on_ is the onset voltage obtained at 1 mW m^–2^.

^*b*^Recorded at 8 V.

^*c*^Maximum external quantum efficiency, followed by data at a current density of 100 mA cm^–2^ and then at 200 mA cm^–2^.

^*d*^Recorded at 15 V.

To further improve the efficiency of NIR-OLEDs, a thin layer (10 nm) of NSeD adjacent to the cathode was added as the electron transporting layer (ETL) to suppress exciton quenching by the cathode while maintaining the total thickness of the OLEDs at 100 nm. The optimized device E (*x* = 50, *y* = 10) achieved a maximum EQE of up to 2.1% (*J* = 42 mA cm^–2^) (Fig. 2d) with an emission at 700 nm. The electroluminescent (EL) spectra are independent of the driving voltage (Fig. S7†). Most notably, the EQE remained almost constant with increasing current densities. At *J* = 100 mA cm^–2^, the value of EQE stayed as high as 2.05%. Even at a higher current density of *J* = 200 mA cm^–2^, the EQE was still 1.96%, which is very desirable for practical application. This indicated that the current-induced exciton quenching was effectively suppressed. On the one hand, as shown in Fig. 2a, the HOMO and LUMO energy levels of NSeD locate right inside those of the host. Such host/guest heterostructures can efficiently suppress the electric field induced quenching and thus minimize the efficiency roll-off in devices.^38^,^49^ On the other hand, both Ga_2_(saph)_2_q_2_ and NSeD have a bipolar character and thus provides a wide charge recombination region.^50^ Overall, the well matched host/guest energy levels and their bipolar characters substantially contribute to the negligible efficiency roll-off of such NIR OLEDs.

In general, the theoretical maximum EQE of OLEDs can be calculated according to the following equation:

1 EQE = *γ* × *η*_r_ × PLQY × *η*_out_

where *γ* is the electron/hole recombination ratio, *η*_r_ is the exciton formation ratio for radiative transitions (*η*_r_ = 0.25 for conventional fluorescent emitters) and *η*_out_ is the light outcoupling efficiency. Given the PLQY of 50 wt% Ga_2_(saph)_2_q_2_:NSeD films of 0.17, the theoretical maximum EQE of device E should be 0.9–1.3%, assuming that *γ* = 1.0, *η*_r_ = 0.25, and *η*_out_ = 0.2–0.3.^51^–^53^ However, the achieved EQE (2.1%) of device E is about 1.62 times as high as the theoretical maximum EQE (1.3%). In fact, all of the other devices with a Ga_2_(saph)_2_q_2_:NSeD blend emitter demonstrated much higher EQEs than their theoretical maximum values.

**Fig. 3:**
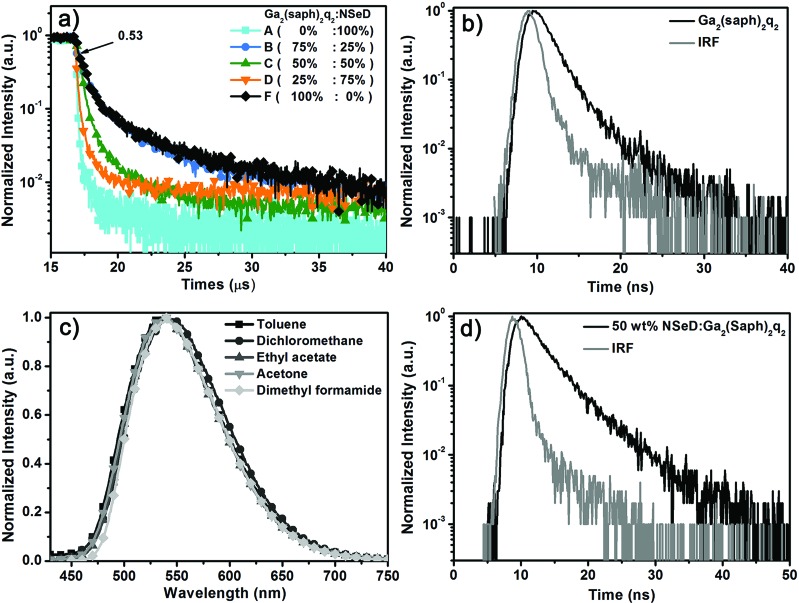
(a) The transient EL decay curves of devices A–D with different Ga_2_(saph)_2_q_2_ concentrations observed at 700 nm with a voltage of 5 V. And the transient EL decay curves of device F (pure Ga_2_(saph)_2_q_2_) observed at 580 nm with a voltage of 5.5 V. (b) The PL transient decay curves of the Ga_2_(Saph)_2_q_2_ film observed at 560 nm. (c) Emission spectra of Ga_2_(Saph)_2_q_2_ in different solvents. (d) The PL transient decay curves of the 50 wt% Ga_2_(saph)_2_q_2_:NSeD film observed at 680 nm (*λ*_ex_ = 380 nm).

To unravel the EL mechanism in these devices, the transient EL measurements were carried out on these devices. It is worth noting that devices A–D have the same device structure, but different ratios of Ga_2_(saph)_2_q_2_ in the EML. Device A with pure NSeD showed a very sharp EL decay curve (Fig. 3a), indicating that almost all of the radiative excitons are short-lived. In contrast, devices B–D with Ga_2_(saph)_2_q_2_:NSeD blend EMLs all demonstrated the existence of delayed fluorescence. Interestingly, the intensity of the delayed component increased with increasing Ga_2_(saph)_2_q_2_ concentration, which suggests that the delayed fluorescence could be correlated with Ga_2_(saph)_2_q_2_. To further identify the origin of the delayed fluorescence, device F with pure Ga_2_(saph)_2_q_2_ as the EML was fabricated with a configuration of ITO/NPB (50 nm)/TCTA (10 nm)/Ga_2_(saph)_2_q_2_ (40 nm)/Mg:Ag (150 nm). As expected, device F showed prominent delayed fluorescence (Fig. 3a) and the delayed fluorescence component contributed about 53% of the total EL signal. However, the transient PL decay of the pure Ga_2_(saph)_2_q_2_ film exhibited a very short lifetime of 1.9 ns (Fig. 3b). Ga_2_(saph)_2_q_2_ showed no solvatochromic effects in different solvents with an emission at 540 nm (Fig. 3c), indicating that the excited singlet state of Ga_2_(saph)_2_q_2_ is not charge-transfer-featured. In addition, the *T*_1_ energy of Ga_2_(saph)_2_q_2_ was found to be 1.96 eV (Fig. S8†), which is about 0.34 eV lower than the *S*_1_ energy (2.30 eV). Accordingly, it could be ruled out that the delayed fluorescence comes from the TADF of Ga_2_(saph)_2_q_2_. On the other hand, the 50 wt% Ga_2_(saph)_2_q_2_:NSeD blend film actually produced a very similar PL spectrum to that of the pure NSeD film and a very short lifetime of 3.4 ns (Fig. 3d). Given the embedded HOMO and LUMO of NSeD are within those of Ga_2_(saph)_2_q_2_, there is no considerable driving force to allow the possible exciplex between Ga_2_(saph)_2_q_2_ and NseD.^54^,^55^ Hence it also could be ruled out that the delayed fluorescence comes from the possible exciplex formed between Ga_2_(saph)_2_q_2_ and NSeD. Therefore, the strong delayed EL could be unambiguously ascribed to extra radiative singlet excitons generated *via* the efficient TF of Ga_2_(saph)_2_q_2_ and then transferred into the singlet state of NSeD, which help bring about the breakthrough of the theoretical maximum EQE in device E.

**Fig. 4:**
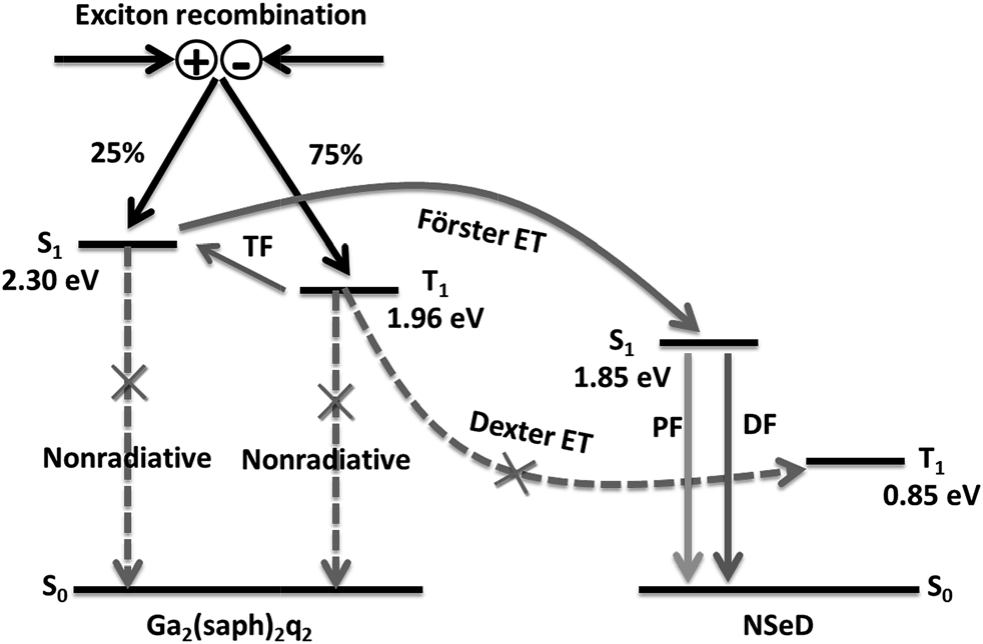
Schematic illustration of the energy transfer processes in NIR-OLEDs. TF stands for triplet fusion, PF for prompt fluorescence, DF for delayed fluorescence and ET for energy transfer. The energy levels given are for Ga_2_(saph)_2_q_2_ and NSeD.

Based on the above results and discussions, we proposed the possible energy transfer processes in these NIR-OLEDs. As shown in Fig. 4, the desirable processes are indicated by solid arrows while the undesirable ones resulting in a loss in efficiency are indicated by dashed arrows with a cross. After charge recombination, the generated singlet exicitons of Ga_2_(saph)_2_q_2_ are directly transferred into the singlet state of NSeD *via* Förster energy transfer and then decay as the prompt fluorescence of NSeD. The triplet excitons of Ga_2_(saph)_2_q_2_ can be up-converted into its singlet states through TF and then transfer into the singlet state of NSeD and decay as the delayed fluorescence of NSeD or, they may Dexter transfer to the triplet state of NSeD, which is a source of loss. It is worth noting that Dexter energy transfer is a short-range coherent transfer of an exciton from a donor to an acceptor site at a rate proportional to the orbital overlap of the donor and acceptor molecules, attenuating exponentially with distance.^26^ In our case, the HOMO and LUMO of NSeD are mainly located on the central naphthoselenadiazole core, which is well protected by the lateral bulky and twisted aryl substituents. These bulky aryl substituents result in a large distance of 9.88–11.30 Å between the neighboring naphthoselenadiazole cores in single crystals (Fig. S9†), which would effectively separate Ga_2_(saph)_2_q_2_ and NSeD molecules in the mixed films and limit their frontier orbital overlap. In addition, the Dexter transfer rate is also proportional to the spectral overlap of the two species because the donor exciton energy must closely match that of the acceptor.^26^ For NSeD, *T*_1_ was calculated to be 0.85 eV, which is much lower than that of Ga_2_(saph)_2_q_2_ (1.96 eV). Such a large energy mismatch (1.11 eV) would inhibit the Dexter energy transfer between the *T*_1_ of Ga_2_(saph)_2_q_2_ and NSeD. Therefore, fortunately, the undesirable Dexter energy transfer between Ga_2_(saph)_2_q_2_ and NSeD can be significantly suppressed. On the other hand, the emission of Ga_2_(saph)_2_q_2_ shows good overlap with the absorption of NSeD (Fig. 1c), which helps provide highly efficient Förster energy transfer between the *S*_1_ of Ga_2_(saph)_2_q_2_ and NSeD. Such efficient Förster energy transfer as well as the efficient TF of Ga_2_(saph)_2_q_2_ contribute to the unexpectedly high EQEs in these NIR-OLEDs with a Ga_2_(saph)_2_q_2_:NSeD blend emitter.

## Conclusions

In summary, high-performance NIR-FOLEDs *via* TF have been achieved with a *R*_max_ of 2127 μW cm^–2^, a maximum EQE of up to 2.1% and the EQE staying at around 2% over a wide range of current densities from 18 to 200 mA cm^–2^, which is amongst the highest performance for NIR-OLEDs based on organic fluorescent materials.^10^,^29^,^46^–^48^ The transient PL and EL studies revealed that such high EQEs (*vs.* the predicted theoretical maximum efficiency of 1.3%) could be attributed to the effective TF from the bipolar host Ga_2_(saph)_2_q_2_. The NIR emitter NSeD, featuring a non-D–A structure and very large HOMO/LUMO overlap, displays a strong deep-red to NIR fluorescence and unique ambipolar character, thus contributing to such unexpectedly high EQEs and negligible efficiency roll-off. It is anticipated that this work provides a promising strategy to realize high-efficiency and low efficiency roll-off NIR-FOLEDs *via* TF.

## Experimental section

### General methods

All commercially available reagents and chemicals were used without further purification. All reactions involving air-sensitive reagents were carried out under an atmosphere of nitrogen. Ga_2_(saph)_2_q_2_, 4,9-dibromonaphtho[2,3-*c*][1,2,5]selenadiazole and [4-(2,2-diphenylvinyl)phenyl]boronic acid were synthesized according to the literature.^36^,^56^,^57^ ^1^H NMR spectra were measured on a JEOLAL-600 MHz spectrometer at ambient temperature. High resolution mass spectra were recorded using a Waters GCT Premier time-of-flight mass spectrometer in electron-impact (EI) mode. Elemental analyses were performed on a flash EA 1112 spectrometer. Absorption spectra were recorded using a UV-vis spectrophotometer (Agilent 8453). The emission spectra and the transient photoluminescence measurements were carried out using a transient spectrometer (Edinburg FL920P). The photoluminescence quantum efficiencies were measured using an absolute photoluminescence quantum yield measurement system (Hamamatsu C11347). The time-resolved PL spectra were measured on a laser flash photolysis spectrometer (Edingburg LP-920). A 1 × 10^–5^ M solution was used for photoluminescence spectra measurements. Small-molecule organic films for optical measurements were fabricated through thermal evaporation under high vacuum (10^–4^ Pa) onto clean quartz substrates. The organic film used for the measurement of the phosphorescence spectra was fabricated through dropping the mixed solution (2%wt Ga_2_(saph)_2_q_2_:10%wt FIrpic: PMMA) onto clean quartz substrates. The ultraviolet photoelectron spectroscopy measurements were performed using a photoelectron spectrometer (Thermo Fisher ESCALAB 250Xi) equipped with a He-discharge lamp providing He–I photons of 21.22 eV with a bias of –5.0 V and the position of the Fermi edge was calibrated using clean Ag film.

### Single-crystal structure

The single crystal of NSeD was obtained in the process of vacuum sublimation. Low temperature (104.6 K) single-crystal X-ray experiments were performed on a Rigaku RAXIS-SPIDER IP diffractometer with graphite-monochromatized Mo_Kα_ radiation (*λ* = 0.71073 Å). Data collection and reduction, cell refinement, and the experiential absorption correction for all compounds were performed with the Rigaku RAPID AUTO software package (Rigaku, 1998, Version 2.30). The structure was solved using direct methods and refined against F2 using full-matrix least-squares techniques. CCDC ; 1402824 contains the supplementary crystallographic data for this paper.†

### Synthesis of NSeD

4,9-Dibromonaphtho[2,3-*c*][1,2,5]selenadiazole (0.98 g, 2.5 mmol), [4-(2,2-diphenylvinyl)phenyl]boronic acid (1.88 g, 6.25 mmol), tetrakis(triphenylphosphine)palladium(0) (0.29 g, 0.25 mmol), potassium carbonate (1.73 g, 12.5 mmol), toluene (50 mL), ethanol (38 mL) and distilled water (25 mL) were first mixed. The mixture was heated to reflux under a nitrogen atmosphere for 24 h. After cooling to room temperature, the mixture was filtered off and washed with hot water, hot petroleum ether and hot ethanol subsequently and dried under vacuum. The crude product was then purified through vacuum train sublimation to afford a red solid (0.87 g, 1.17 mmol, 47%). ^1^H NMR (600 MHz, CD_2_Cl_2_, *δ*): 7.731 (m, 2H), 7.449–7.296 (m, 24H), 7.247 (d, *J* = 8.1 Hz, 4H), 7.186 (m, 2H), 7.139 (s, 2H). HRMS-EI (*m*/*z*): calcd for C_50_H_34_N_2_Se, 742.1887; found: 742.1871. Elemental analysis calcd for C_50_H_34_N_2_Se: C, 80.96; H, 4.62; N, 3.78; found: C, 81.10; H, 4.65; N, 3.90.

### Theoretical calculations and computational details

All of the calculations were performed using the Gaussian 09 program package.^58^ The ground state geometries of NSeD were optimized based on the single-crystal structure. The triplet energy of NSeD was calculated based on the optimized ground-state geometry. Calculations on the ground and excited electronic states of NSeD were performed with density functional theory (DFT) and time-dependent DFT (TDDFT) using the B3LYP functional with the 6–31G(d) basis set in a vacuum.^59^,^60^

### Device fabrication and measurements

The devices were fabricated through thermal evaporation under high vacuum (*ca.* 7 × 10^–4^ Pa) onto ITO-coated glass substrates. The substrates were carefully cleaned and treated with UV ozone for 10 min before vacuum thermal deposition. All OLED devices were encapsulated in a standard dry nitrogen glove box after fabrication and then measured under ambient conditions. The measurements of the transient electroluminescence were carried out using a transient spectrometer (Edinburg FL920P) and an Agilent 8114A Programmable Pulse Generator was used to generate appropriate excitation waveforms with a pulse width of 15 μs. The amplitude of the pulse was 8 V or 8.5 V, and the baseline was –3 V. The period was 50 μs, delay time 25 μs, and duty cycle 30%. The current–voltage characteristics were measured with a Keithley 4200 semiconductor characterization system and the optical power was determined using a Newport 1936-C power meter coupled to a calibrated Newport 918D-UV-OD3 detector with a spectral response range from 200 to 1100 nm. The electroluminescence spectra of the OLEDs were obtained using a multichannel spectrometer (PR 705). The EQE of the NIR EL was determined according to the literature method, by measuring the light intensity in the forward direction and assuming the external emission profile to be Lambertian.^61^,^62^

## Supplementary Material

Supplementary information

Crystal structure data
