# Supplementary material for: High-efficiency and low efficiency roll-off near-infrared fluorescent OLEDs through triplet fusion
Source: Chem Sci. 2016 Jan 19;7(4):2888–95. doi: 10.1039/c5sc04685h (PMC6054030; doi:10.1039/c5sc04685h)
Supplement: Supplementary file 1 [file SC-007-C5SC04685H-s001.pdf]

Electronic Supplementary Material (ESI) for Chemical Science.
This journal is © The Royal Society of Chemistry 2016

Supporting Information

High-efficiency and Low Efficiency Roll-Off Near-Infrared Fluorescent OLEDs through Triplet Fusion

Jie Xue, Chen Li, Lijun Xin, Lian Duan* and Juan Qiao*

Key Lab of Organic Optoelectronics and Molecular Engineering of Ministry of Education, Department of Chemistry, Tsinghua University, Beijing 100084, P R China

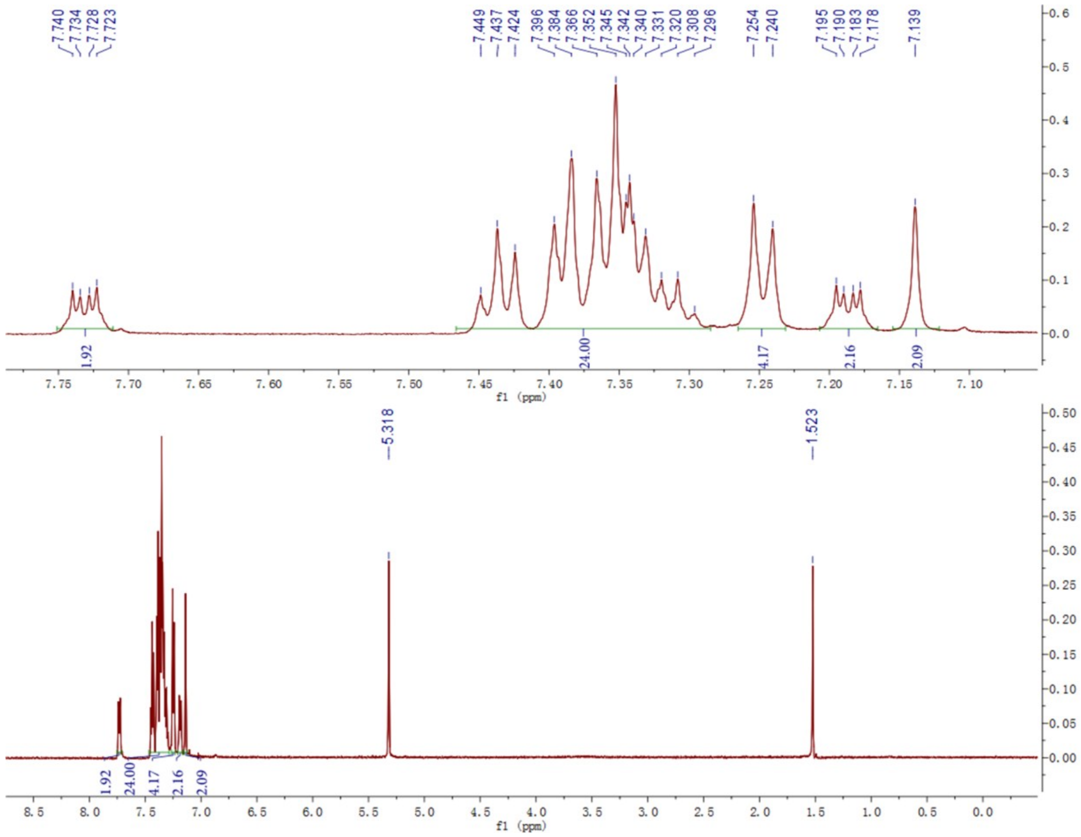

Fig. S1 ^{1}H NMR spectrum of NSeD.

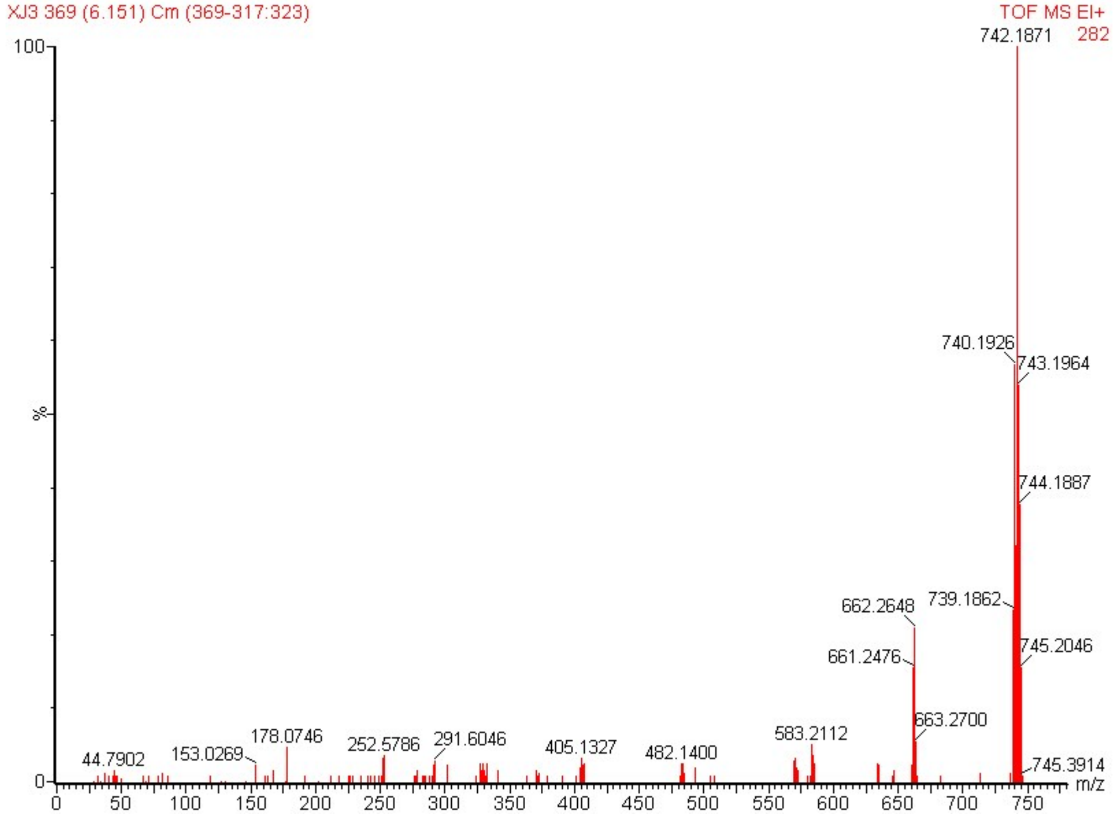

Fig. S2 HRMS-EI Mass spectrum of NSeD.

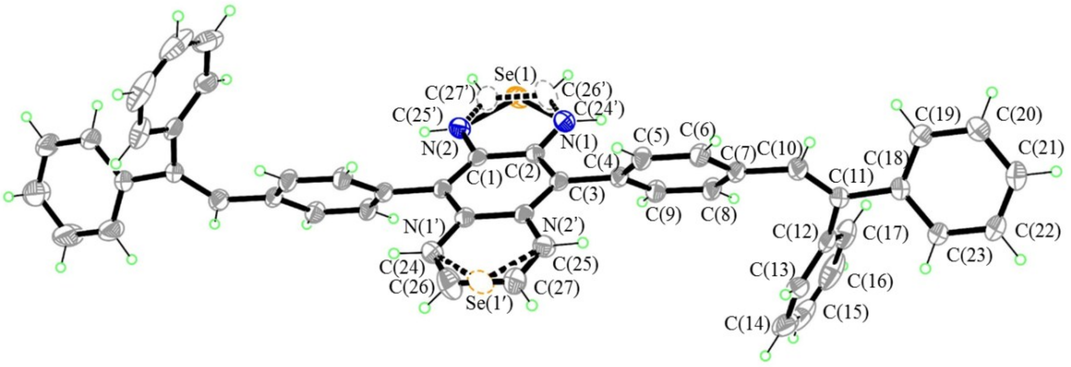

Fig. S3 Single crystal structure of NSeD with thermal ellipsoids plotted at 50% probability level.

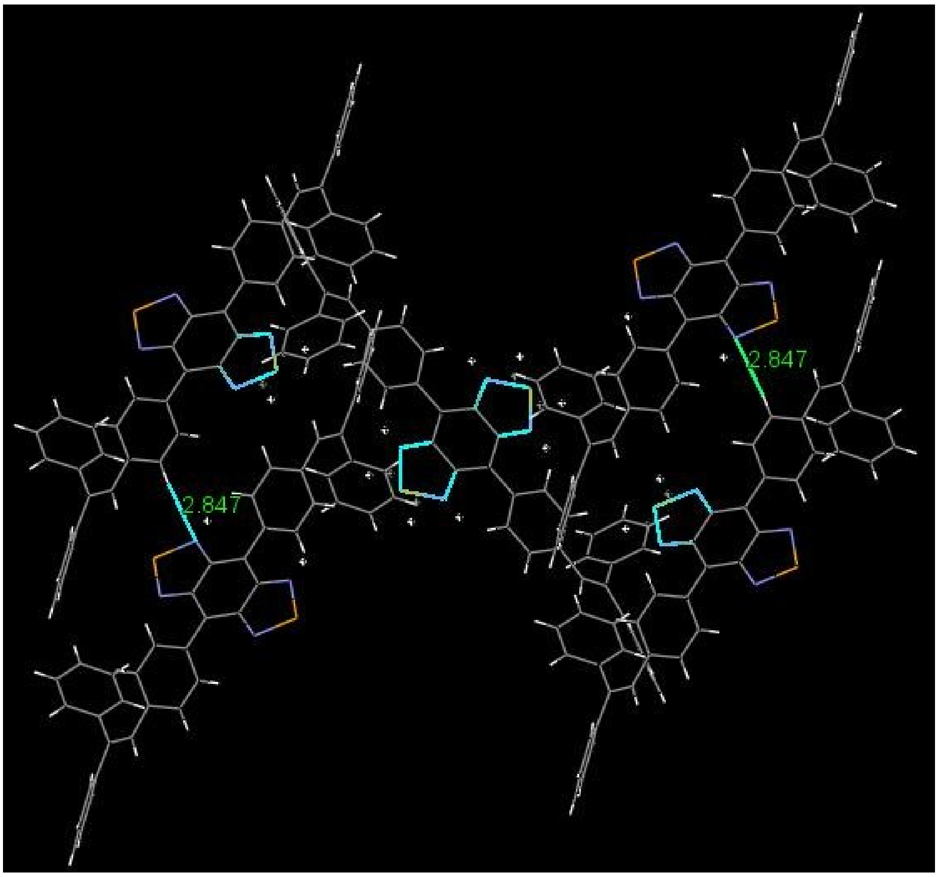

Fig. S4 Molecular packing diagram of NSeD in single crystal with denoted intermolecular interactions.

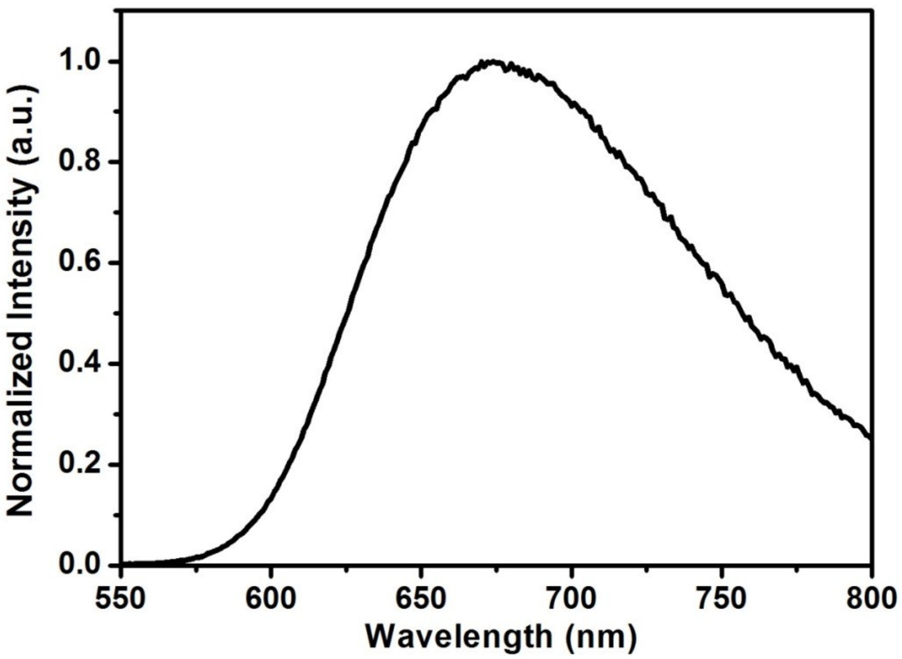

Fig. S5 The emission spectrum of NSeD in toluene solution at 298K.

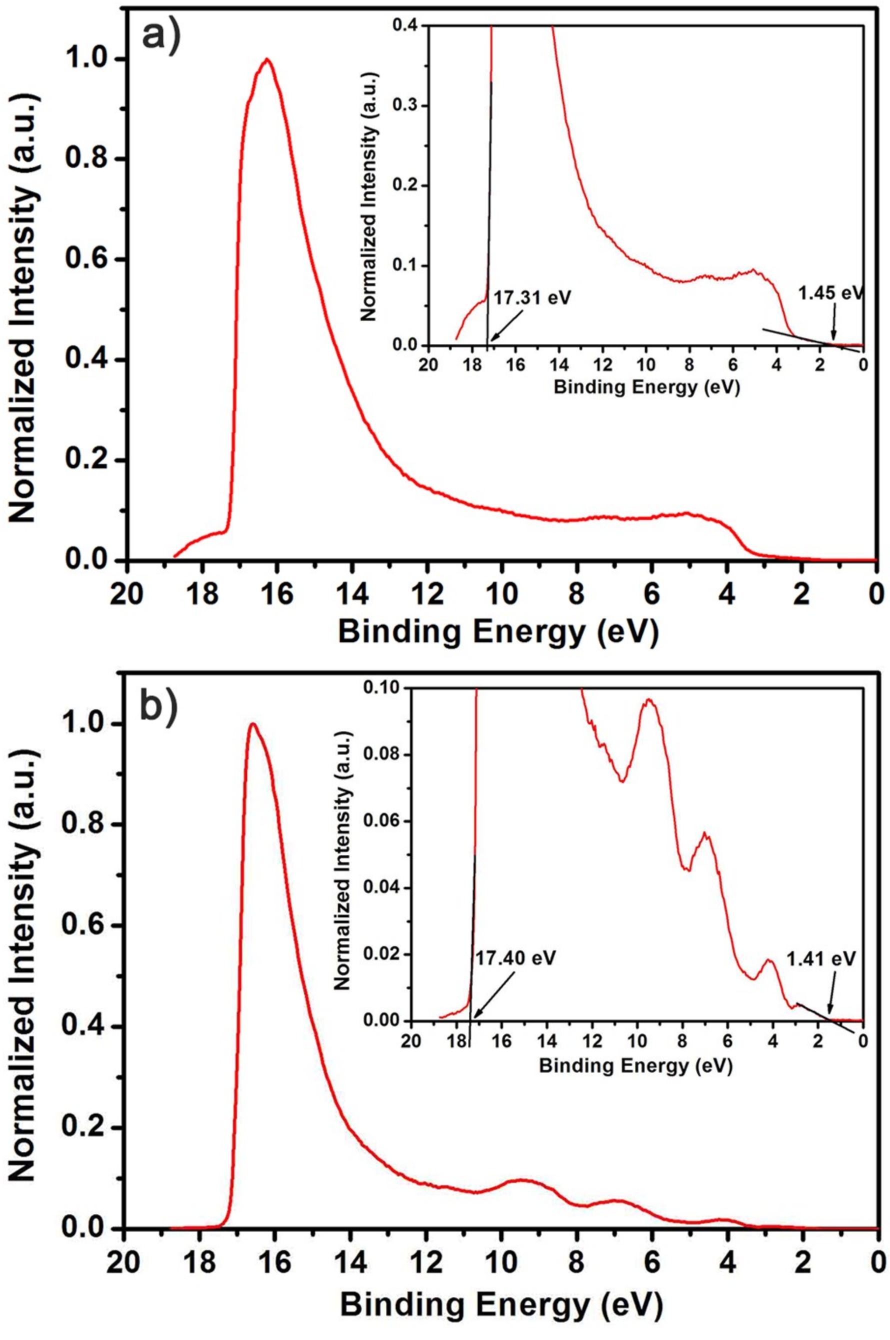

Fig. S6 The ultraviolet photoelectron spectroscopy spectra of (a) $Ga_2(saph)_2q_2$ and (b) NSeD. Inset: zoom-in view of the cutoff and HOMO regions.

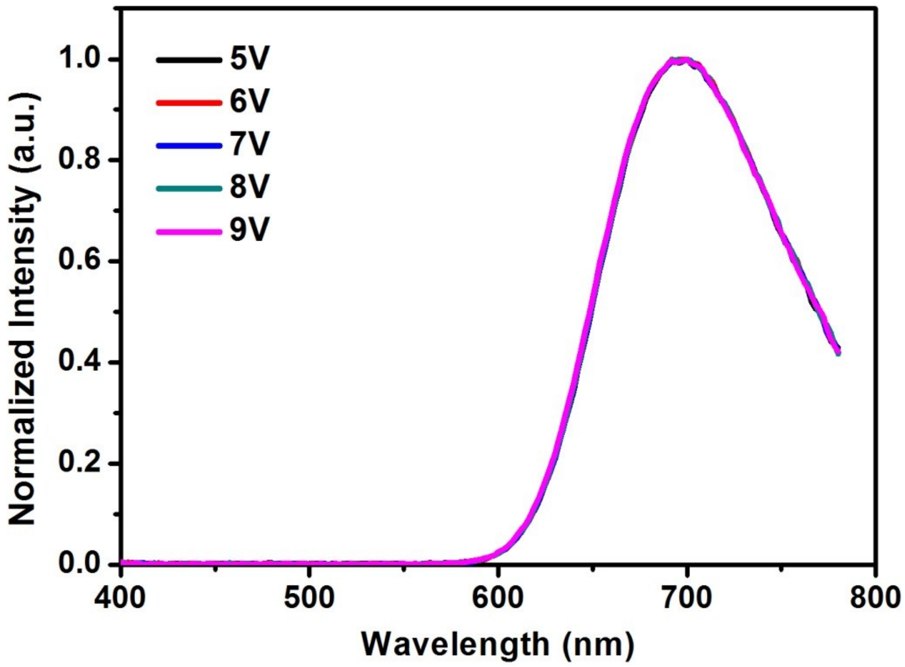

Fig. S7 Electroluminescent spectra of device E recorded at various voltages.

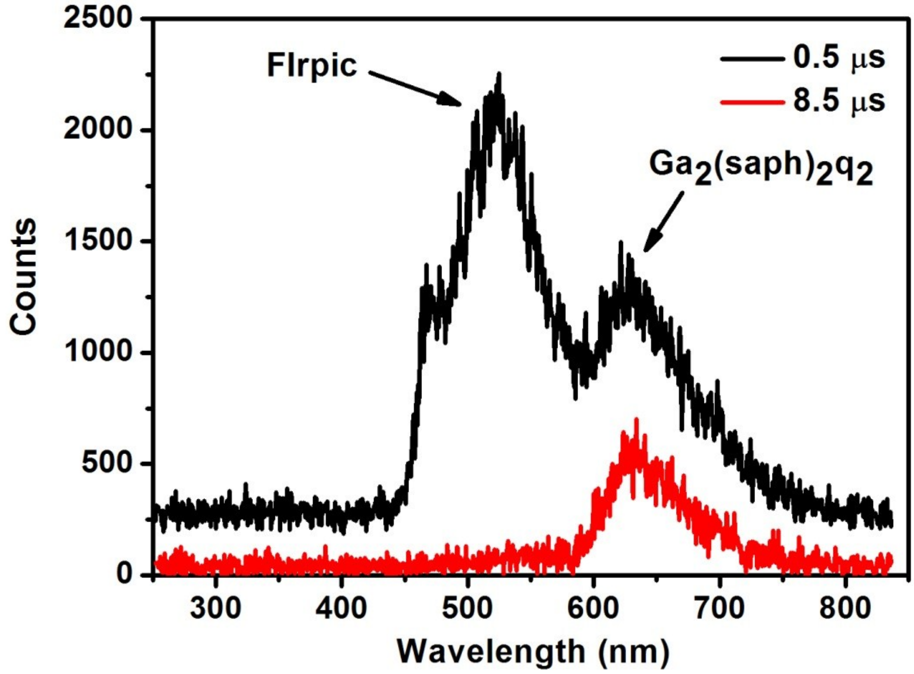

Fig. S8 Phosphorescence spectra of $Ga_2(saph)_2q_2$ doped in PMMA host with FIrpic triplet sensitizer. The spectra were recorded with different delayed time scales after excitation (λ_{ex} = 355 nm) for the $Ga_2(saph)_2q_2$ doped film.

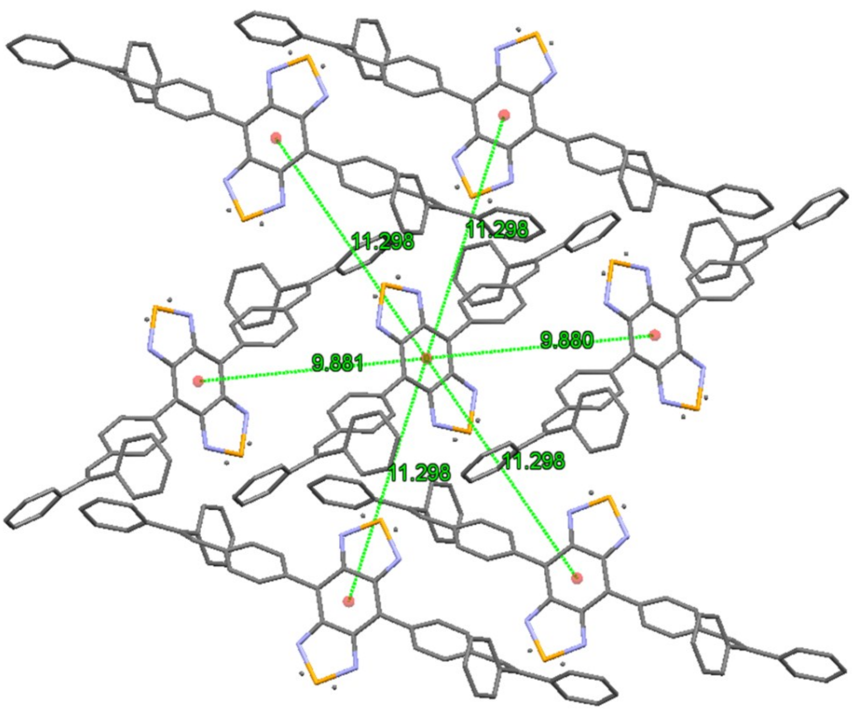

Fig. S9 Partial crystal packing diagram of NSeD with denoted distance between naphthoselenadiazole cores.
